# Outcome after neoadjuvant chemotherapy in elderly breast cancer patients – a pooled analysis of individual patient data from eight prospectively randomized controlled trials

**DOI:** 10.18632/oncotarget.24586

**Published:** 2018-02-26

**Authors:** Gabriel von Waldenfels, Sibylle Loibl, Jenny Furlanetto, Anna Machleidt, Bianca Lederer, Carsten Denkert, Claus Hanusch, Sherko Kümmel, Gunter von Minckwitz, Andreas Schneeweiss, Michael Untch, Kerstin Rhiem, Peter A. Fasching, Jens-Uwe Blohmer

**Affiliations:** ^1^ Department of Gynecology and Breast Center, Charité University Hospital, Berlin, Germany; ^2^ German Breast Group, Neu-Isenburg, Germany; ^3^ Institute of Pathology, Charité University Hospital, Berlin, Germany; ^4^ Rotkreuzklinikum, Munich, Germany; ^5^ Breast Center, Kliniken Essen-Mitte, Essen, Germany; ^6^ National Center for Tumor Diseases (NCT), Heidelberg, Germany; ^7^ HELIOS Klinikum Berlin-Buch, Berlin, Germany; ^8^ Center for Hereditary Breast and Ovarian Cancer, University Hospital Cologne, Köln, Germany; ^9^ Department of Gynecology and Obstetrics, University Hospital Erlangen, Comprehensive Cancer Center Erlangen-EMN, Friedrich-Alexander University Erlangen-Nuremberg, Erlangen, Germany

**Keywords:** elderly, pCR, neoadjuvant, breast, cancer survival, Gerotarget

## Abstract

**Introduction:**

Recent studies showed the high and independent impact of age (<40 years) on pathologic complete response (pCR) and prognosis for patients undergoing neoadjuvant chemotherapy (NACT). Some physicians might not consider elderly patients (>65 years) for NACT due to poor prognosis or higher toxicity. The aim of this analysis is to help selecting appropriately elderly women who would benefit from NACT. Secondly, survival parameters are investigated in several histological subgroups.

**Methods:**

From 1998 to 2010, eight prospectively randomized German Breast Group (GBG) trials of anthracycline- and taxane-based NACT were performed and analyzed in this study.

**Results:**

Compared to the overall average, elderly women had significant larger tumors and more overall lymph node involvement. Histologically, they had more G2 tumors, more estrogen-receptor positive tumors. pCR (ypT0 ypN0) was strongly associated with age. The multivariable logistic regression analysis of clinical parameters showed that young age, clinical stage T4, invasive ductal cancer and poor differentiated breast cancer are predictive for high pCR. The multivariate analyses of molecular subgroups showed that age >65years is a predictor of significant lower pCR in HER2**−** breast cancers. Nonetheless, HER2+ patients showed pCR rates as high**−** and HR+/HER2+ even higher - pCR rates compared to younger patients.

**Discussion:**

This study underlines the unfavorable impact of higher age on pCR, but it shows a realistic chance for pCR if NACT is applied - especially for HER2+ patients. Furthermore, elderly patients with non-TNBC showed a good prognosis (comparable to younger patients) regarding overall survival, even if they do not have pCR.

## INTRODUCTION

Age is considered a primary risk factor for the development of breast cancer. It is foreseen that, in the coming decades, approximately 20% of the population will be aged over 65 years and therefore, it is expected that the proportion of older women with breast cancer will grow considerably. Recent studies suggest that the median age for breast cancer diagnosis is approximately 60 years, and over 40% of all breast cancers diagnosed are in women aged 65 years or older [1]. In Germany a survey showed that more than half of all breast cancer patients are older than 65 years and more than a third are older than 70 years [2].

Management of older breast cancer patients is challenging. In general, chemotherapy is less often used in elderly [3], but the relative benefit from chemotherapy is independent from age [4]. The neoadjuvant use of chemotherapy seems even less of an option, partly due to the fact that it is less investigated in this population [3]. For some physicians neoadjuvant chemotherapy is only an option for elderly patients with inflammatory breast cancer or with locally advanced inoperable breast cancer. This might withhold the advantages of NACT, like prediction of prognosis and response adapted therapy, from elderly patients [5]. A recent analysis [6] of eight GBG-trials showed the high and independent impact of age on pathologic complete response (pCR) as well as the association of age with prognosis for patients undergoing NACT. Some other studies report as well that women >65 years have a lower pCR rate and detrimental prognosis as well as a higher toxicity compared to younger women [7, 8].

The first aim of this analysis is to help selecting appropriately elderly women who would benefit from NACT. Furthermore, we want to assess the effect of age on disease free survival (DFS), local-recurrence-free survival (LRFS), distant disease free survival (DDFS) and overall survival (OS) in the overall group, in the pCR group, in the non-pCR group and in biological subgroups defined by hormone receptor and HER2-status.

The hypothesis is that elderly breast cancer patients (>65 y) with specific histological characteristics might have a high pCR rate or a good prognosis even without pCR after NACT compared to younger patients (<40 y).

## MATERIALS AND METHODS

### Patients

From 1998 to 2010, eight prospectively randomized GBG-trials investigating NACT, GeparDuo [9], GeparTrio pilot [10] and main study [11], GeparQuattro [12], AGO 1 [13], Prepare [14], Techno [15], GeparQuinto trial [16] were performed. Patient data of these studies were combined in a metadatabase analysis. All trials had the approval of the responsible ethics committee. All patients gave written consent for their participation and data collection. The eight trials had comparable eligibility criteria [17]: All patients needed to have a primary breast cancer measurable either by ultrasound, palpation or mammography. Diagnosis was histologically confirmed by core biopsy. The tumor size had to be ≥ 1 cm in the ultrasound in GeparQuattro and GeparQuinto, ≥ 3 cm in AGO1 and ≥ 2 cm in all other trials. ER/PR status was defined as positive if at least 10 % of cells stained positive. The NACT of each trial contained an anthracycline- and taxane-based chemotherapy backbone. In GeparQuattro and Techno, HER2+ patients received one year of trastuzumab. In GeparQuinto, HER2+ patients received trastuzumab and lapatinib versus bevacizumab if HER2−, with non-responders after four cycles of epirubicin and cyclophosphamide being randomized to a treatment with paclitaxel with or without everolimus. We included all patients with minimum one cycle of preoperative medical treatment in this analysis. Patients with estrogen receptor (ER) and/or progesterone receptor (PR) positive breast cancer had to receive adjuvant endocrine treatment for at least 5 years according to German guidelines [18]. Also, adjuvant radiation therapy had to be performed accordingly (e.g. whole breast radiation therapy after breast conserving surgery or radiation of thoracic wall after mastectomy for clinical and/or histological stages T3, T4, N2, N3) [18].

### Objectives and endpoints

The primary aim of this combined analysis was to identify elderly patients likely to profit from NACT by evaluating pCR rates for women >65 y compared to younger patients (age groups: <40 y; 40–50 y; 51–65 y), overall and in different biological subgroups defined by hormone receptor (HR) and HER2-status. Pathological complete response was defined as ypT0 ypN0 (no invasive and no non-invasive residuals in the breast and nodes) [5].

Further aims were to assess the effect of age >65 y on DFS, LRFS, DDFS and OS, overall, in the pCR- and non-pCR group and in biological subgroups compared to younger patients.

### Statistics

Individual data at surgery and in follow-up was extracted for this combined analysis from all participating 8949 patients. As defined in the protocols, patients with missing data on histopathological response, e.g. because of having no surgery, were counted as having no response. Baseline parameters were correlated with pCR using two-sided Pearson Chi square test or Fisher’s exact test. Survival was calculated by the date of randomization to event or last follow-up and plotted as Kaplan–Meier curves with log-rank *p*-*value*s. Odds ratios and hazard ratios, 95% confidence intervals (CI) and corresponding *p*-*value*s between categorized score values were calculated using logistic regression and Cox regression analysis. No adjustment was made for performing multiple tests, and all of the probability values were two sided with an alpha of 0.05 for statistical significance. SPSS 20.0 was used to perform analyses.

## RESULTS

From the total of 8949 patients included in the analysis 566 (6.3%) were older than 65 years. Median age of all patients was 49 years (21–80 y). Older patients had a significantly higher body mass index (BMI) compared to younger patients. Information about ER, PR, HER2 was available in 6763 (75.6%) patients. Table 1 describes the baseline characteristics of patients, tumor and surgery.

**Table 1:** Baseline characteristics of patients, tumor and surgery

	<40 y *n* = 1453		40−50 y *n* = 3420		51−65 y *n* = 3510		>65 y *n* = 566		all patients *n* = 8949		*p* value^*^
***n***	**Valid %**	***n***	**Valid %**	***n***	**Valid %**	***n***	**Valid %**	***n***	**Valid %**
**Tumor stage**											<0.001
cT1	135	9.4	283	8.3	192	5.5	24	4.3	634	7.1	
cT2	984	68.3	2189	64.5	2175	62.4	310	55.3	5658	63.7	
cT3	214	14.9	586	17.3	567	16.3	95	16.9	1462	16.5	
cT4a-c	50	3.5	181	5.3	277	8.0	67	11.9	575	6.5	
cT4d	57	4.0	153	4.5	272	7.8	65	11.6	547	6.2	
Missing									73	0.8	
**Nodal status**											0.031
N0	731	51.6	1670	49.7	1649	47.9	249	44.5	4299	49.0	
N 1−3	619	43.7	1518	45.2	1578	45.8	272	48.6	3987	45.4	
N 4−9	50	3.5	131	3.9	163	4.7	32	5.7	376	4.3	
N >10	18	1.3	38	1.1	56	1.6	7	1.3	119	1.4	
Missing									168	1.9	
**Histological type**											<0.001
Ductal invasive	1221	85.7	2721	81.5	2720	79.0	439	77.6	7101	81.0	
Lobular invasive	86	6.0	400	12.0	502	14.6	78	13.8	1066	12.2	
Others	117	8.2	217	6.5	221	6.4	49	8.7	604	6.9	
Missing									178	2.0	
**Tumor grade**											<0.001
G1	42	3.1	137	4.2	119	3.6	17	3.1	315	3.7	
G2	684	49.8	1812	55.6	1839	55.6	322	59.1	4657	54.9	
G3	647	47.1	1312	40.2	1350	40.8	206	37.8	3515	41.4	
Missing									462	5.2	
**ER status**											<0.001
Negative	630	45.4	1244	37.8	1206	35.8	198	35.2	3278	38.1	
Positive	758	54.6	2050	62.2	2160	64.2	365	64.8	5333	61.9	
Missing									338	3.8	
**PR status**											<0.001
Negative	700	50.5	1415	43.0	1611	48.0	284	50.5	4010	46.6	
Positive	687	49.5	1878	57.0	1746	52.0	278	49.5	4589	53.4	
Missing									350	3.9	
**HER-2 Status**											0.072
Negative	829	72.1	2050	74.4	1974	74.2	371	78.4	5224	74.2	
Positive	320	27.9	707	25.6	688	25.8	102	21.6	1817	25.8	
Missing									1908	21.3	
**Molecular subtypes**											<0.001
HR positive/HER2−	463	42.3	1309	49.2	1319	51.8	256	54.9	3347	49.5	
HR positive/HER2+	174	15.9	419	15.8	358	14.1	54	11.6	1005	14.9	
HR negative/HER2+	137	12.5	275	10.3	313	12.3	48	10.3	773	11.4	
TNBC	320	29.3	655	24.6	555	21.8	108	23.2	1638	24.2	
Missing									2186	24.4	
**BMI**											<0.001
<18.5	42	2.9	63	1.9	30	0.9	3	0.5	138	1.6	
18.5−24.9	895	62.0	1802	53.1	1377	39.7	189	33.4	4263	48.0	
25.0−29.9	325	22.5	1009	29.7	1224	35.3	215	38.0	2773	31.2	
30.0−39.9	163	11.3	480	14.1	784	22.6	145	25.6	1572	17.7	
≥40	19	1.3	40	1.2	55	1.6	14	2.5	128	1.4	
Missing									75	0.8	
**Surgery Type**											<0.001
Breast conserving therapy	992	71.9	2321	70.7	2333	70.1	310	58.5	5956	69.9	
Mastectomy	387	28.1	964	29.3	997	29.9	220	41.5	2568	30.1	
Missing									425	4.7	

* *χ*^2^ or Fisher´s test.

Women older than 65 years had significant larger tumors stage T4a-d, lymph node involvement (LN 1–9) compared to the other age groups and lobular invasive tumors compared to patients less than 50 years. Histologically, the majority of patients > 65 years had G2 and estrogen receptor positive tumors. Compared to the young women (<40 years) there is no difference for PR. HER2-status was not statistically different between the age groups. There were more HR+/HER2− tumors and fewer HR+/HER2+ tumors in the elderly group compared to all other patient groups. Women >65 years had less triple negative tumors than women <40 years. The rate of HR−/HER2+ tumors was between 10.3% and 12.5% in all groups. HR–/HER2+ tumors were the most rarely found subtype in our cohort with 11.4%.

### pCR analyses

We found an age dependent rate of pCR, which showed that pCR is lowest for elderly >65 patients (11.7%) and is higher with decreasing age. The highest pCR rate is found in the group of <40 year old women (20.9%) (Table 3).

**Table 3:** pCR

Age	pCR (%)	Logistic regression^*^ Odds Ratio (95% CI)	*p*-value
**overall**			
>65	11.7	1.00	
51–65	14.1	1.32 (0.99–1.77)	0.063
40–50	17.3	1.57 (1.18–2.10)	0.002
<40	20.9	1.84 (1.35–2.50)	<0.001
**HR+/HER2−**			
>65	3.1	1.00	
51–65	6.2	2.06 (0.98–4.32)	0.058
40–50	8.3	2.70 (1.29–5.65)	0.008
<40	11.0	3.45 (1.59–7.46)	0.002
**HR+/HER2+**			
>65	20.4	1.00	
51–65	19.0	0.90 (0.40–1.95)	0.786
40–50	18.4	0.87 (0.40–1.88)	0.716
<40	19.0	0.88 (0.38–2.03)	0.756
**HR−/HER2+**			
>65	33.3	1.00	
51–65	34.8	1.13 (0.56–2.25)	0.737
40–50	29.1	0.81 (0.40–1.65)	0.562
<40	29.2	0.94 (0.44–2.02)	0.877
**TNBC**			
>65	19.4	1.00	
51–65	26.5	1.50 (0.88–2.56)	0.136
40–50	35.6	2.07 (1.23–3.50)	0.007
<40	38.8	2.21 (1.27–3.84)	0.005

* adjusted for study, tumor and nodal stage, histotype, grading.

In the HR+/HER− group women >65 years had lowest pCR rates of all subgroups (3.1%).

Within the two HER2+ subgroups, no significant age related differences were found.

For TNBC, the pCR rate was also significantly the lowest in the subgroup of women >65 years.

The multivariable logistic regression analysis of clinical parameters showed that young age, clinical stage T4a-d, invasive ductal cancer and G3 breast cancer are predictive for high pCR. The multivariate analyses of molecular subgroups also showed that age >65years is a predictor of significant lower pCR in TNBC, HR+/HER2–, G3 and N+ breast cancers.

### Survival analyses

During a median follow up period of 62.5 (62.0−63.0) months, 717 (8.0%) local-recurrences, 1799 distant events (20.1%), and 1336 (14.9%) deaths were observed.

**Figure 1:**
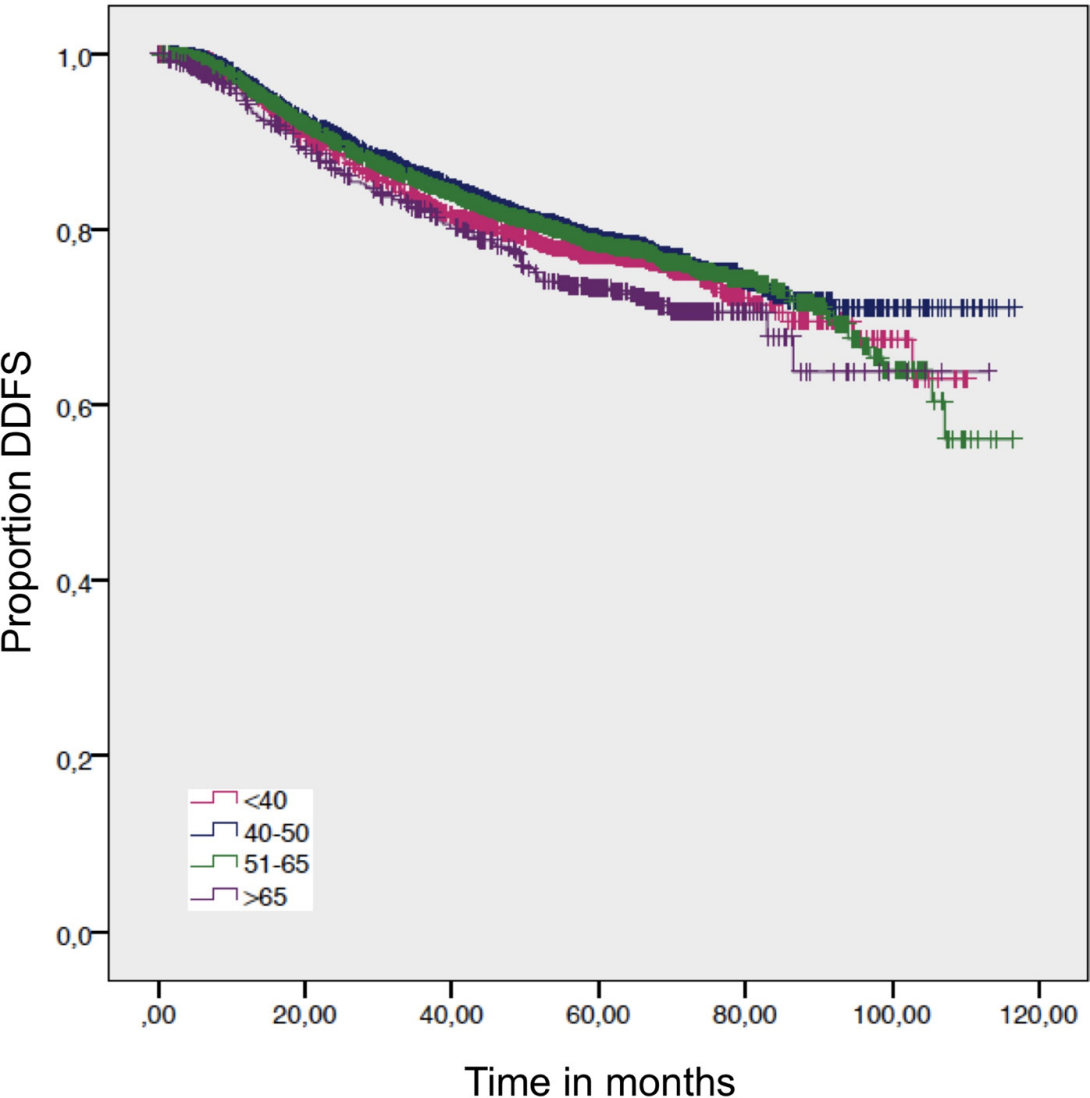
Distant disease free survival by age Log-rank *p*-value = 0.016.

Women >65 years had a significant better LRFS than women between 40-50 years and women <40 years (Table 2; Figure 1).

**Table 2:** MVA cox regression of survival data

Age	DDFS				LRFS				DFS				OS			
**HR**	**95%CI**		***p* value**	**HR**	**95%CI**		***p* value**	**HR**	**95%CI**		***p* value**	**HR**	**95%CI**		***p* value**
**All subtypes**																
>65	1.00															
51–65	0.85	0.70	1.02	0.082	1.21	0.85	1.72	0.287	0.90	0.75	1.08	0.267	0.73	0.59	0.90	0.003
40–50	0.87	0.72	1.06	0.164	1.44	1.01	2.05	0.043	0.96	0.80	1.16	0.696	0.74	0.60	0.91	0.005
<40	1.02	0.82	1.25	0.882	1.95	1.34	2.82	<0.001	1.16	0.95	1.41	0.153	0.86	0.68	1.09	0.202
**pCR**																
No pCR	1.00															
pCR vs no pCR	0.38	0.32	0.45	<0.001	0.33	0.25	0.44	<0.001	0.40	0.34	0.47	0.058	0.71	0.47	1.07	0.105
**HR positive/HER2 negative**																
>65	1.00															
51–65	0.76	0.55	1.03	0.080	0.89	0.48	1.66	0.714	0.77	0.57	1.05	0.096	0.60	0.42	0.86	0.005
40–50	0.71	0.52	0.98	0.038	1.13	0.61	2.09	0.706	0.80	0.59	1.09	0.154	0.50	0.35	0.73	<0.001
<40	0.98	0.69	1.41	0.924	1.71	0.88	3.30	0.113	1.10	0.78	1.54	0.600	0.66	0.43	1.01	0.057
**pCR**																
No pCR	1.00															
pCR	0.43	0.28	0.68	<0.001	0.60	0.32	1.15	0.124	0.51	0.34	0.75	0.001	0.34	0.18	0.64	0.001
**HR positive/HER2 positive**																
>65	1.00															
51–65	1.01	0.50	2.01	0.989	4.02	0.54	30.20	0.177	1.10	0.55	2.18	0.795	0.56	0.25	1.28	0.169
40–50	1.13	0.57	2.25	0.730	6.20	0.84	45.88	0.074	1.40	0.71	2.76	0.325	0.83	0.37	1.85	0.649
<40	1.10	0.52	2.35	0.803	6.82	0.89	52.44	0.065	1.44	0.70	2.99	0.323	0.51	0.19	1.34	0.172
**pCR**																
No pCR																
pCR	0.66	0.41	1.06	0.086	1.03	0.57	1.87	0.912	0.78	0.51	1.17	0.223	0.41	0.19	0.90	0.026
**HR negative/HER2 positive**																
>65	1.00															
51–65	1.87	0.86	4.10	0.117	1.92	0.58	6.33	0.282	1.94	0.93	4.03	0.077	1.37	0.58	3.24	0.469
40–50	2.33	1.06	5.13	0.036	2.21	0.66	7.36	0.197	2.38	1.14	4.99	0.021	1.60	0.67	3.81	0.290
<40	1.84	0.79	4.27	0.157	2.30	0.66	8.02	0.194	1.78	0.80	3.94	0.155	1.46	0.58	3.70	0.425
**pCR**																
No pCR																
pCR	0.25	0.16	0.40	<0.001	0.14	0.06	0.33	<0.001	0.23	0.15	0.36	<0.001	0.18	0.09	0.35	<0.001
**TNBC**																
>65	1.00															
51–65	0.98	0.68	1.41	0.917	1.55	0.86	2.81	0.145	1.18	0.83	1.67	0.359	0.94	0.63	1.39	0.741
40–50	1.19	0.83	1.70	0.353	1.72	0.96	3.11	0.071	1.33	0.94	1.88	0.113	1.14	0.77	1.68	0.511
<40	1.01	0.68	1.51	0.952	1.76	0.94	3.29	0.075	1.22	0.83	1.78	0.309	1.05	0.68	1.61	0.826
**pCR**																
No pCR																
pCR	0.18	0.13	0.25	<0.001	0.12	0.07	0.21	<0.001	0.18	0.13	0.24	<0.001	0.17	0.12	0.24	<0.001

**Figure 2:**
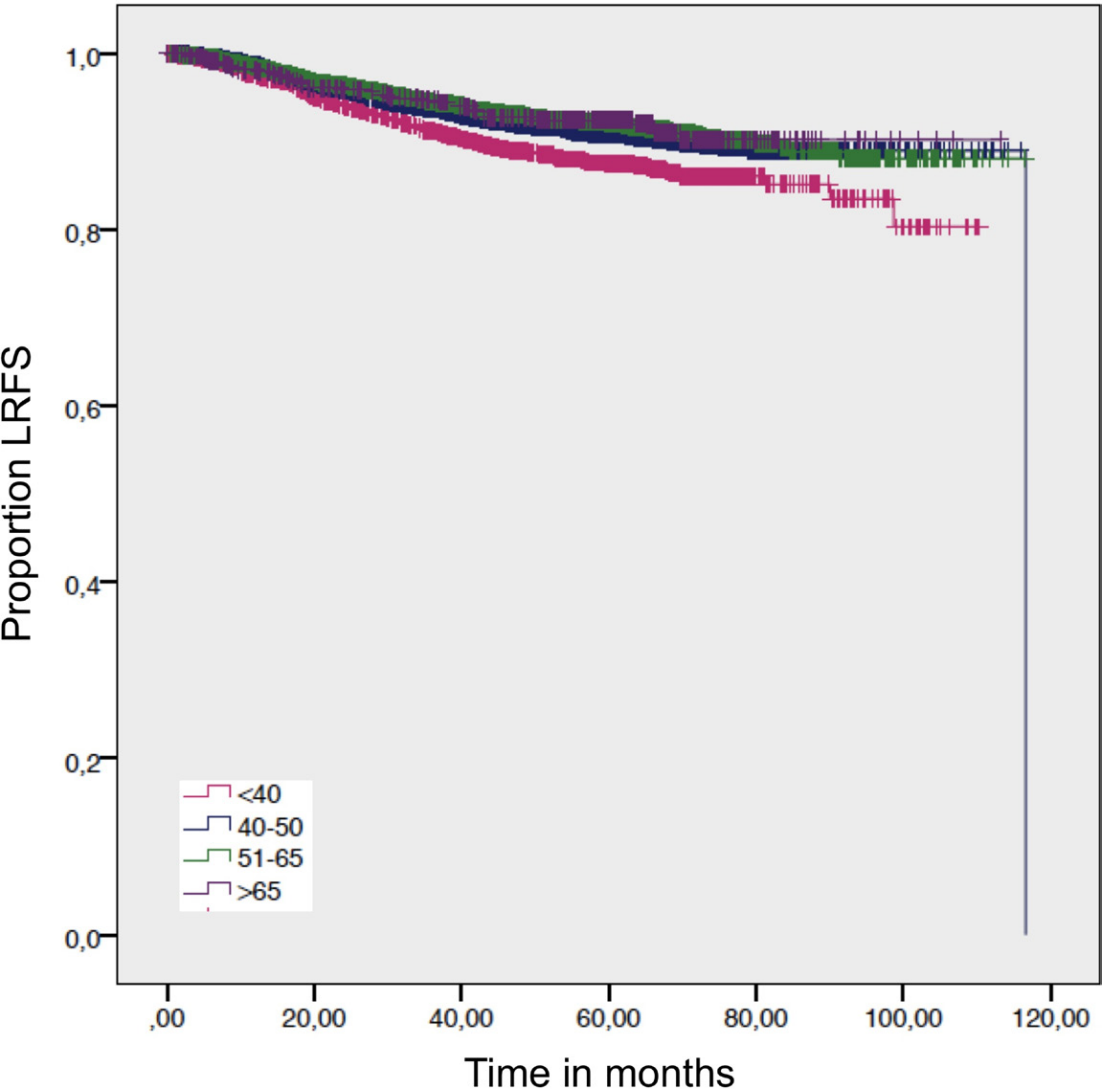
Local-recurrence-free survival by age Log-rank *p*-value = 0.001.

**Figure 3:**
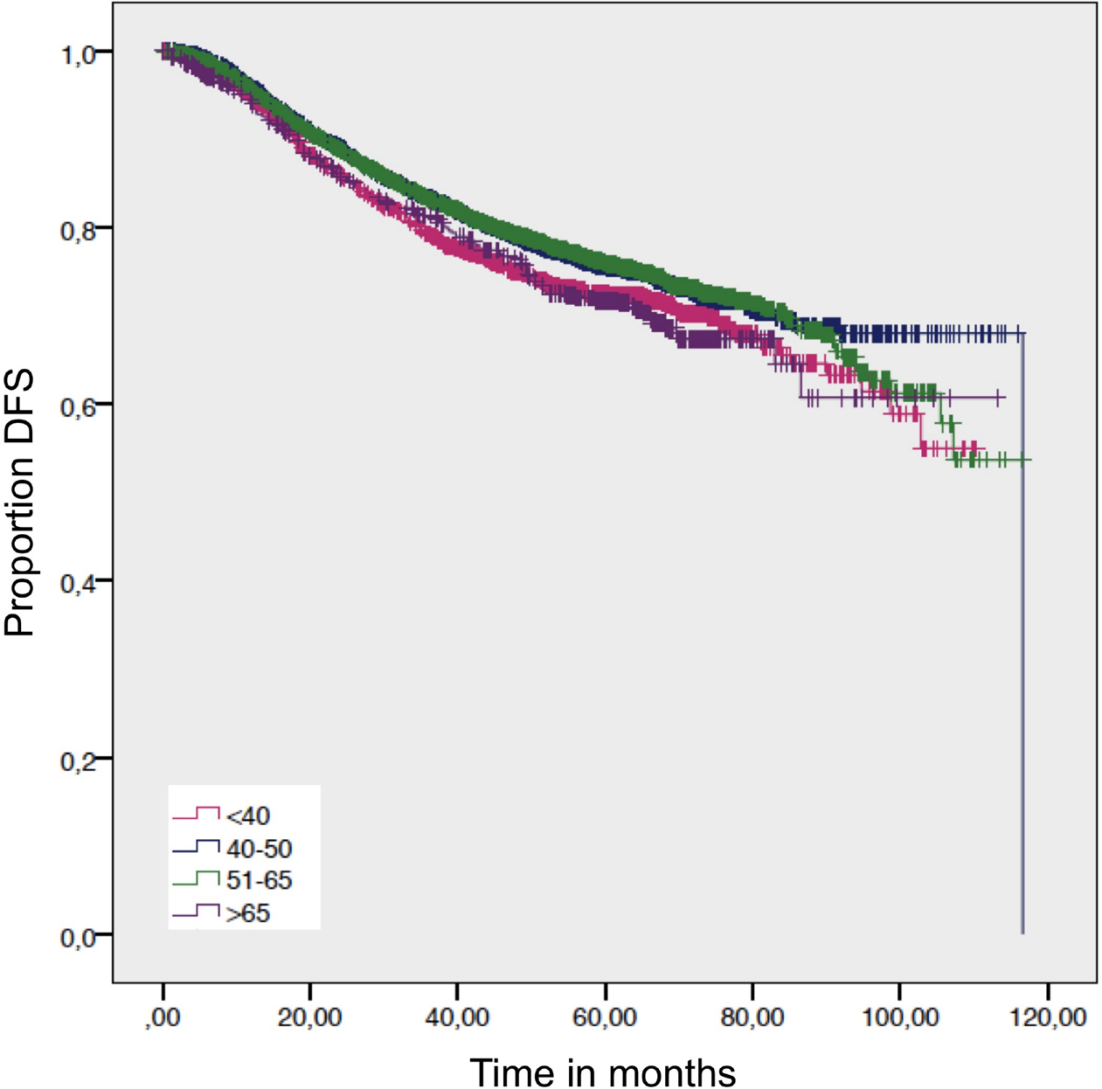
Disease free survival Log-rank *p*-value = 0.009.

For the overall survival, we found a significantly worse outcome for patients >65 years compared to women age 51–65 years and women 40–50 years. The DDFS and DFS – which exclude death from other factors than cancer - showed no statistical difference (Figure 2; Figure 3; Table 2). Despite not showing statistical significance, women >65 years had a worse DFS compared to women 40–50 y and 51–65 years, but not compared to women <40 years (Figure 3).

Within the breast cancer subtypes, a significant better DDFS was found for patients between 40−50 years old compared to the elderly >65 in the HR+/HER2− group. In the HR−/HER2+ subgroup the DDFS for the same group of age was significantly worse compared to the elderly >65 years. (Table 2).

**Figure 4:**
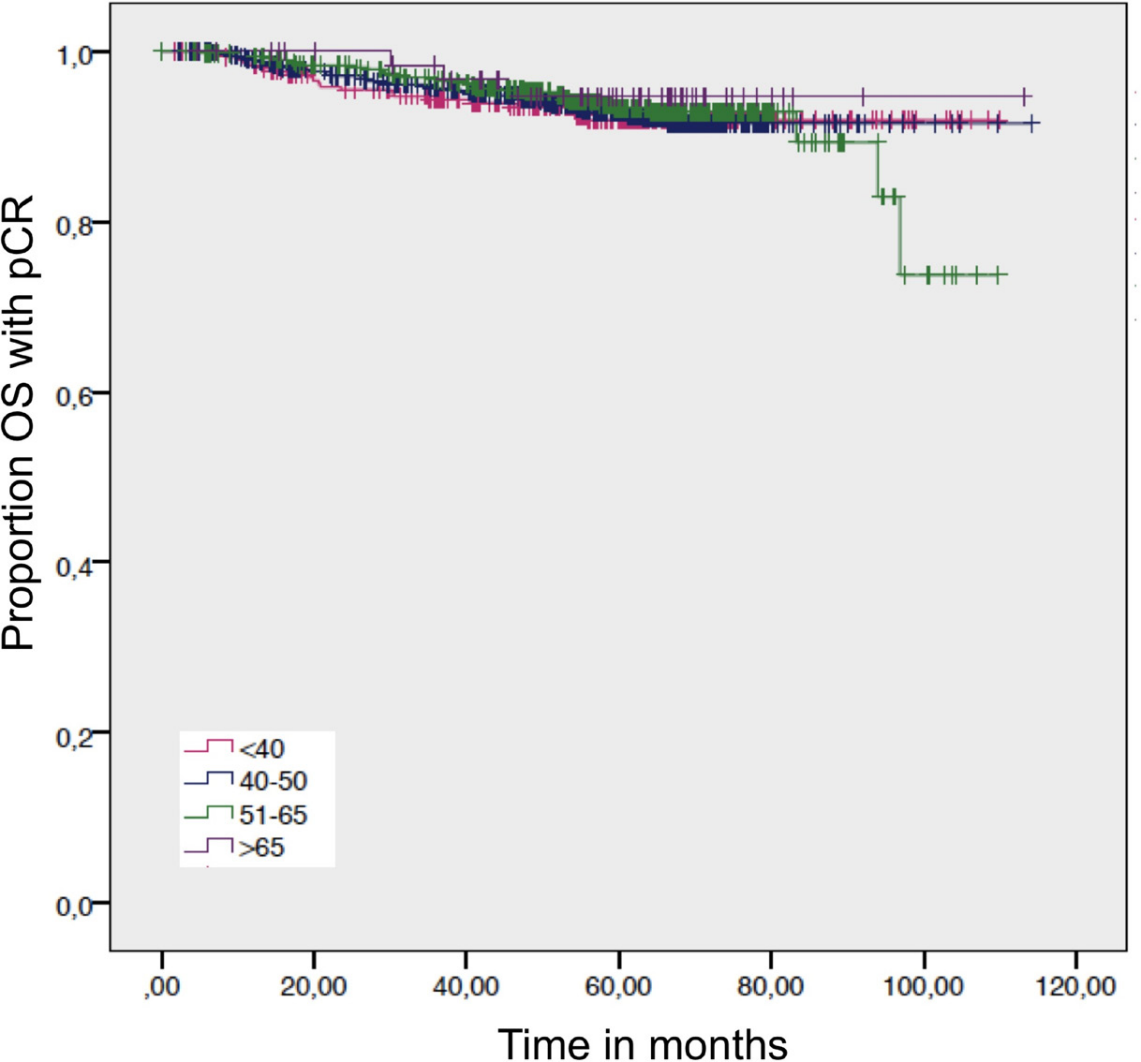
Overall survival in patients with a pathological complete response by age Log-rank *p*-value = 0.899.

**Figure 5:**
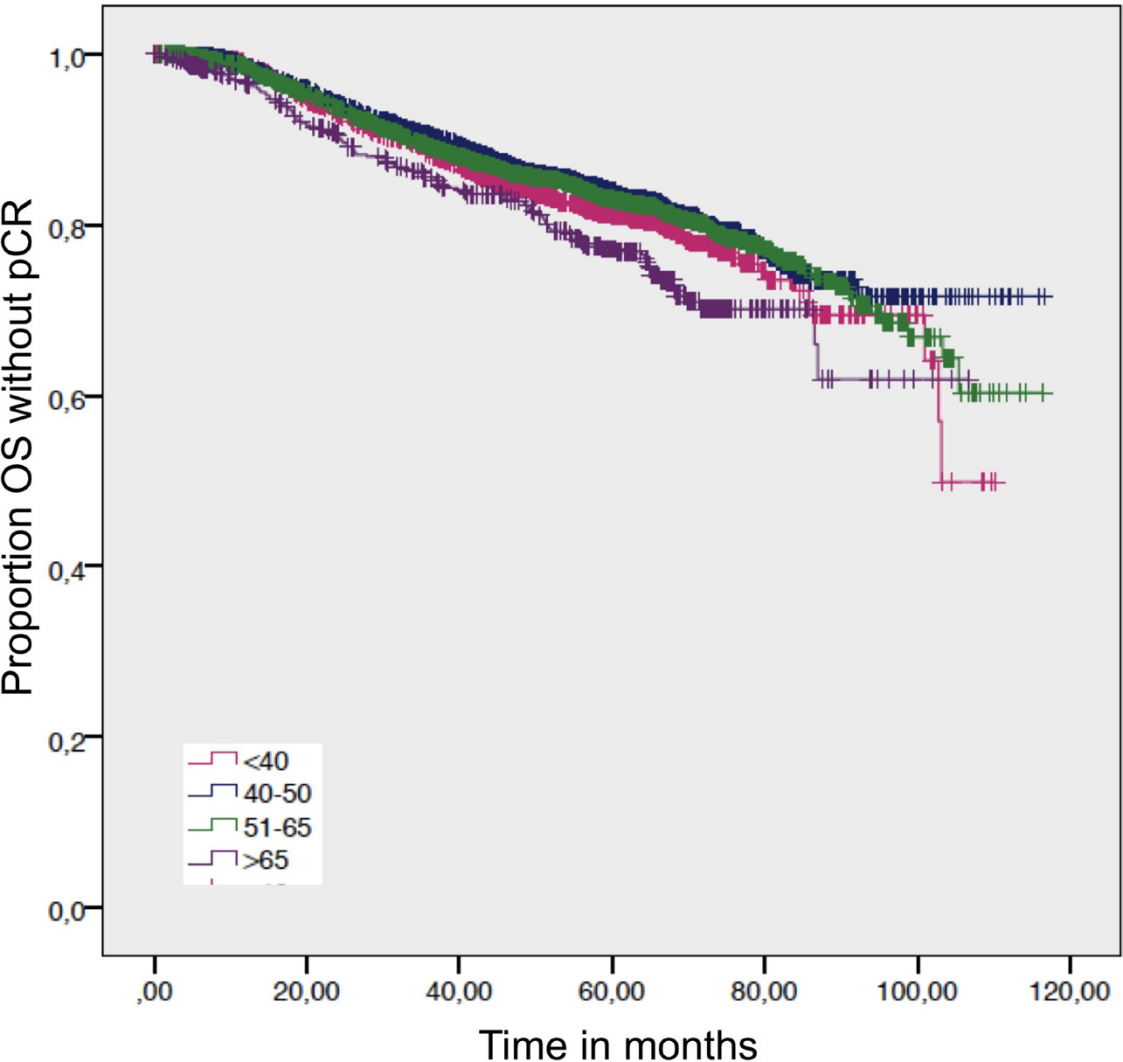
Overall survival in patients without a pathological complete response by age Log-rank *p*-value = 0.001.

A statistical significance for OS was evaluated for the HR+/HER2– subgroup, where the elderly >65 had a survival disadvantage compared to women age 51−65 years and women 40−50 years (Table 2). This survival disadvantage (OS) for women >65 years is not sustained when a pCR is achieved (Figures 4 and 5).

**Figure 6:**
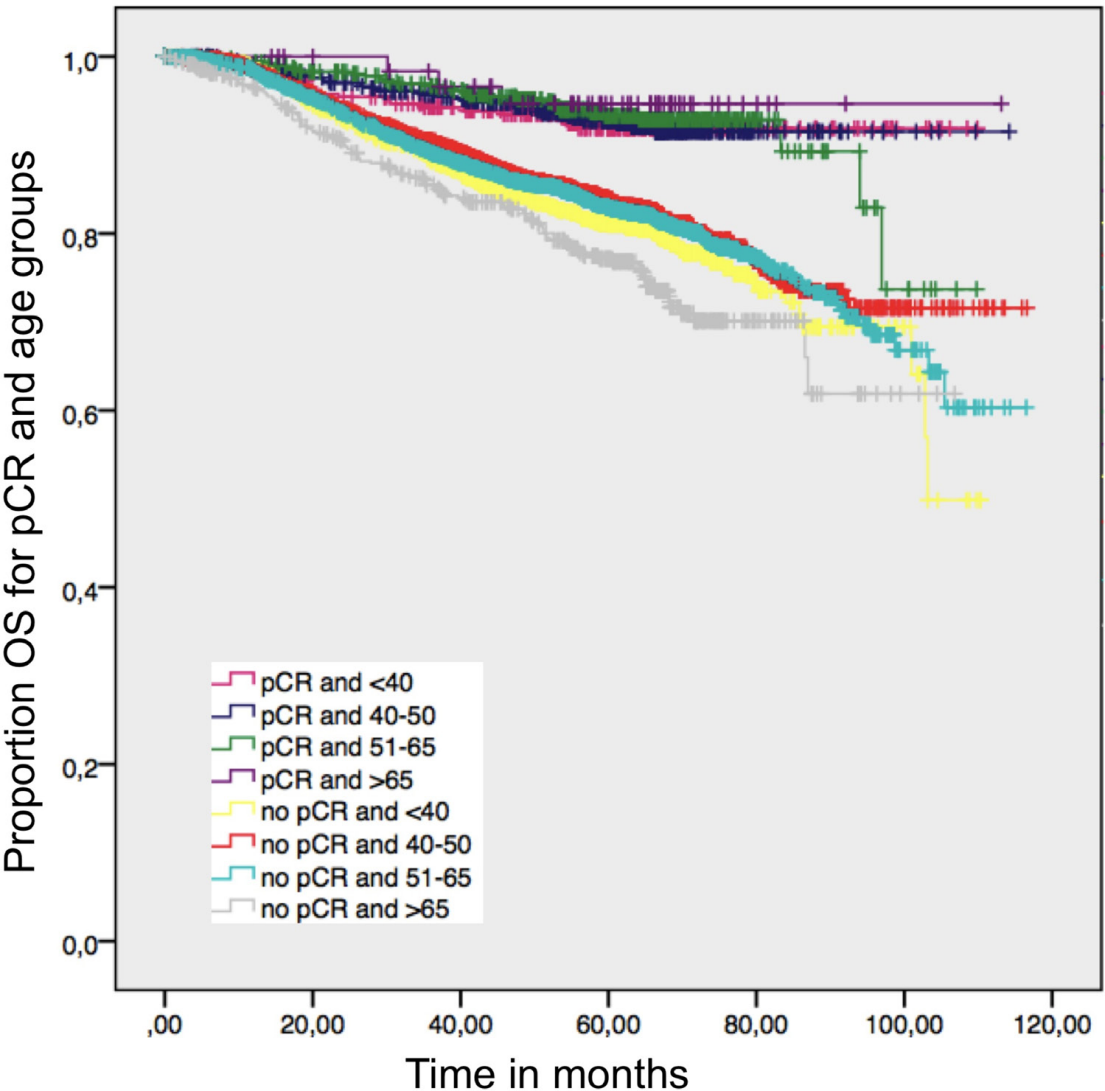
Overall survival in patients with and without a pathological complete response by age; log-rank *p* value = 0.001

pCR is strongly associated with better OS in all age groups (Figure 6). There were no significant differences between age >65 years and other age groups if pCR was achieved.

Besides age, pCR, N-status and tumor stage had significant impact on DFS, LRFS, DDFS, and OS. Additionally, the grading had a significant impact on DFS, LRFS and OS but not on DDFS.

After adjustment for known prognostic factors and age, women >65 years had a worse OS compared to the age group 40−50 (HR = 0.74 [95% CI 0.60−0.91]; *p* = 0.005) and 51–65 (HR = 0.73 [95% CI 0.59−0.90]; *p* = 0.003). Elderly women with HR+/HER2– subtype had a significantly worse OS compared to patients aged 40–50 (HR = 0.50 [95% CI 0.35–0.73]; *p* < 0.001) and 51–65 (HR = 0.60 [95% CI 0.42–0.86]; *p* = 0.005). No statistically significant difference was found in the other subgroups.

## DISCUSSION

Half of the women with breast cancer in Germany are older than 65 years [2]. This is not only a numerous but also important group in need for treatment - especially as the life expectancy in Germany and western European countries is rising [19]. In our analysis of 8949 patients the percentage of women >65 years is only 6.32%. This shows again that elderly are underrepresented in clinical trials [20].

It has to be mentioned critically, that there is no information about Ki-67 activity in this analysis to distinguish Luminal A from Luminal B tumors, which benefit differently from chemotherapy [18]. Another limitation of this study is that we do not have information about residual cancer burden or tumor-infiltrating lymphocytes which are identified as predictors of chemotherapy response in other studies [21, 22]. The analyzed database does not provide information about chemotherapy related toxicity. For every patient, the relation between risk and benefit of an anticancer therapy has to be evaluated before and during treatment. Elderly patients often have more comorbidities and often receive comedication. According to a systematical review [23] there are age related differences in pharmacokinetics of breast cancer treatment as they contain anthracyclines (reduced clearance) and platinum agents (reduced creatinine clearance) – as in all NACT protocols of this study. But it is still questionable whether these differences have any clinical relevance [23]. In general, age cannot be seen as an absolute determinant for the prediction of pharmacokinetics of neoplastic agents, as it does not account for organ function entirely. A more practical approach might be to perform more functional geriatric assessments. A BMI ≥30 (minimum obesity grade 1) in the group of elderly >65 was found in 28.1%. Interestingly, in the group of young women <40 years it was only found in 12.6%. Obesity is a known risk factor for complications like coronary heart disease, worse impact on chronic heart failure, diabetes type II and therefore also renal insufficiency – known risk factors for survival outcomes and oftentimes limiting factors for the dosage or even reason for discontinuation of anticancer agents.

The analysis also showed that pCR is not only age dependent, but also differs significantly between the biological subtypes of breast cancer. In the triple negative and HR+/HER2− subgroups the pCR rate of the elderly >65 years is significantly lower in comparison to all other groups. Again, a continuously increasing pCR rate was found for higher age. Nevertheless, this effect was not found in HR+/HER2+ and HR−/HER2+ cohort. The pCR rate was significantly lower in elderly patients with HR+/HER2− and TNBC breast cancer. On the other hand, pCR rates in our analysis were not different between elderly and younger patients in the histological subgroups HR+/HER2+ and HR–/HER2+. As HER2+ specific therapies such as trastuzumab are routinely added to chemotherapy this effect may dominate the age dependent absolute effect of chemotherapy. Age might be negated partially by the effectiveness of anti-HER2 treatment, but it was not available for all patients in this analysis (not available in GeparDuo, GeparTrio, AGO 1 and Prepare).

In addition, DFS, LRFS, DDFS and OS are significantly longer in the group of patients >65 years, if pCR is achieved in the TNBC subtype. For the other subtypes there is no significant difference found. Missing systemic therapy for TNBC besides chemotherapy and in contrast effective systemic therapy for HR+ and HER2+ tumors may be an explanation. These findings support the necessity for the more frequent consideration of neoadjuvant antihormonal treatment for these patients, and is under further investigation in other multicenter trials [24].

The pCR rates do not necessarily correlate with shortened OS in all histological subtypes, as other studies already showed [25, 26].

An explanation for lower pCR may be that the tumors of the elderly, as shown in this population, contain more G2 differentiated tumors and less G3 than all other groups, and also have the highest rate of invasive lobular cancers. These characteristics are unfavorable for pCR, as predictors for achieving pCR rather are G3- and invasive ductal cacinomas [25, 27].

The overall survival of elderly women in our study was worse compared to younger patients. For patients >65 years all prognostic outcomes (DFS, DDFS, LRFS, OS) were associated with immunohistological subtype as shown in other recent publications [28]. The breast cancer related prognosis is not related to a specific age, but it depends on individual tumor associated factors like specific gene expression [29]. Higher prevalence of HR-positive tumors is age related [29]. In our study we only have OS data but no breast cancer specific survival data. J. Patnaik *et al*. showed that comorbidity is associated with decreased OS and increased mortality. Furthermore it was shown that patients >65 years with comorbid condition and stage I tumor had similar or poorer OS compared to patients who had no comorbid condition and stage II tumor [30].

In conclusion, this study supports the data showing the unfavorable impact of age on pCR, especially for TNBC. Nevertheless, it shows the realistic chance of pCR using NACT. Especially pCR rates of HER2+ patients were as high - and for HR+/HER2+ even higher - than of younger patients. Secondly, elderly patients with non-TNBC have a comparably good prognosis regarding OS, even if no pCR is achieved.

## Abbreviations

BMI: body mass index
CI: confidence interval
DDFS: distant disease free survival
DFS: disease free survival
ER: estrogen receptor
LRFS: local-recurrence-free survival
MVA: multivariate analysis
NACT: neoadjuvant chemotherapy
OS: overall survival
pCR: pathological complete response
PR: progesterone receptor
TNBC: triple negative breast candor.
